# Effects of low-dye taping on plantar pressure pre and post exercise: an exploratory study

**DOI:** 10.1186/1471-2474-10-40

**Published:** 2009-04-21

**Authors:** Damien Nolan, Norelee Kennedy

**Affiliations:** 1 Physiotherapy Department, University of Limerick, Limerick, Ireland

## Abstract

**Background:**

Low-Dye taping is used for excessive pronation at the subtalar joint of the foot. Previous research has focused on the tape's immediate effect on plantar pressure. Its effectiveness following exercise has not been investigated. Peak plantar pressure distribution provides an indirect representation of subtalar joint kinematics. The objectives of the study were **1) **To determine the effects of Low-Dye taping on peak plantar pressure immediately post-application. **2) **To determine whether any initial effects are maintained following exercise.

**Methods:**

12 asymptomatic subjects participated; each being screened for excessive pronation (navicular drop > 10 mm). Plantar pressure data was recorded, using the F-scan, at four intervals during the testing session: un-taped, baseline-taped, post-exercise session 1, and post-exercise session 2. Each exercise session consisted of a 10-minute walk at a normal pace. The foot was divided into 6 regions during data analysis. Repeated-measures analysis of variance (ANOVA) was used to assess regional pressure variations across the four testing conditions.

**Results:**

Reduced lateral forefoot peak plantar pressure was the only significant difference immediately post tape application (*p *= 0.039). This effect was lost after 10 minutes of exercise (*p *= 0.036). Each exercise session resulted in significantly higher medial forefoot peak pressure compared to un-taped; (*p *= 0.015) and (*p *= 0.014) respectively, and baseline-taped; (*p *= 0.036) and (*p *= 0.015) respectively. Medial and lateral rearfoot values had also increased after the second session (*p *= 0.004), following their non-significant reduction at baseline-taped. A trend towards a medial-to-lateral shift in pressure present in the midfoot immediately following tape application was still present after 20 minutes of exercise.

**Conclusion:**

Low-Dye tape's initial effect of reduced lateral forefoot peak plantar pressure was lost after a 10-minute walk. However, the tape continued to have an effect on the medial forefoot after 20 minutes of exercise. Further studies with larger sample sizes are required to examine the important finding of the anti-pronatory trend present in the midfoot.

## Background

Low-Dye (LD) taping is an orthopaedic strapping technique of the foot involving the application of tape inferior to the malleoli, and along the plantar aspect of the foot [1]. A variety of different LD taping techniques, all modified from the original method described by Dr. Ralph Dye, have been identified in the literature [2].

Low-Dye taping is used by physiotherapists for the management of symptoms related to excessive pronation at the subtalar joint of the foot [3]. Pronation itself is a functionally important movement, assisting in the medial transfer of weight across the foot during the stance phase of gait. However, excessive pronation occurs when the subtalar joint remains pronated beyond the midstance period of the gait cycle [4]. This excessive pronation can result in increased soft tissue stress and changes in overall lower limb alignment, often predisposing the individual in question to injury of the lower extremity [5]. The LD taping technique aims to reduce excessive pronation by creating an external supinating force medial to the subtalar joint axis, namely at the medial plantar surface of the foot [6]. This helps to raise the medial longitudinal arch, and bring the subtalar joint closer to its neutral position.

One method used to assess whether LD tape is reducing pronation is by measuring changes in plantar pressure, i.e. pressure along the sole of the foot. Although no direct link between plantar pressure and joint motion has been established, it is thought that changes in subtalar alignment during the stance phase of gait lead to subsequent changes in plantar pressure distribution [2]. Research has shown that LD taping has an effect on plantar pressure during gait by promoting anti-pronatory changes in pressure (e.g.; a reduction in pressure along the medial aspect of the foot) in both pronated and normal subtalar joints [2,7]. However, these studies only examined the immediate effects of LD taping, and did not investigate if the LD tape remains effective following a timed period of weight-bearing activity. Lange et al. [2] stated that future research should examine the effect of LD taping on plantar pressure over a longer period of time.

Previous research has investigated the duration of effect of LD taping on other measures of subtalar joint pronation, such as navicular drop [1,6,8] and rearfoot motion [9], but plantar pressure remains unexplored. Therefore, the aims of this study were to firstly determine the effects of LD taping on peak plantar pressure immediately post-application, and, secondly, whether the tape's effects on peak plantar pressure were still being maintained following a timed period of exercise.

## Methods

Ethical approval was obtained from the University of Limerick Research Ethics Committee. Written informed consent was acquired from all subjects.

### Subjects

A convenience sample of 21 healthy subjects volunteered to participate in the study. Subjects were initially screened for eligibility to enter the study using the navicular drop test. This involved measuring the height of the navicular when standing with the foot placed in subtalar neutral (by the investigator), and again when the subject was in relaxed standing [10]. The navicular drop test is regularly used as a method of measuring excessive pronation in healthy subjects, exhibiting good intra-rater reliability [10]. A navicular drop greater than 10 mm was necessary for participation, as this is indicative of excessive pronation [11]. Those with a navicular drop of 10 mm or less were excluded from the study. The right foot was used for taping in subjects with bilateral excessive pronation. Further inclusion criteria for participation included being aged 18 or over, and willing and able to walk independently at a comfortable pace for two 10-minute walking sessions. Exclusion criteria included an injury to the lower limb in the previous six months, gait affected by pain, injury, or neurological condition, a history of lower limb surgery, and a known lower limb pathology (other than pronated feet). Tape allergy testing was also performed during the screening session. This involved applying a strip of zinc oxide tape to the foot, which was left intact for 24 hours. Subjects were excluded from the study if they exhibited any signs of allergy (excessive redness/rash/skin peeling) on removal of the tape.

### Study Design

A single-group repeated measures design was used.

### Instrumentation

**Figure 1:**
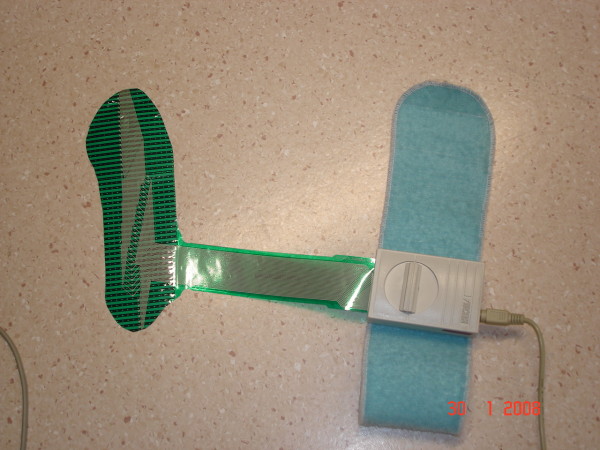
**F-scan components – shown are the plastic insole, Velcro strap, cuff unit, and cable**.

The F-scan™ (Tekscan Inc), a computerized insole sensor system, was used to measure plantar pressure (Figure 1). The F-scan is a bi-pedal system consisting of a thin (0.18 mm) plastic insole to measure pressure distribution at the foot-shoe interface via 960 individual pressure-sensing points located on the insole. The insole is attached to a cuff unit (98 × 64 × 29 mm), which itself is then attached to the individual's leg by means of a Velcro strap. A 9.25 m-long cable connects the cuff unit to a computer, which contains the "Tekscan F-scan Clinical TAM version 5.72" software to record the plantar pressure measurements. The F-scan has been shown to exhibit moderate to good reliability [12,13]

### Taping technique

**Figure 2:**
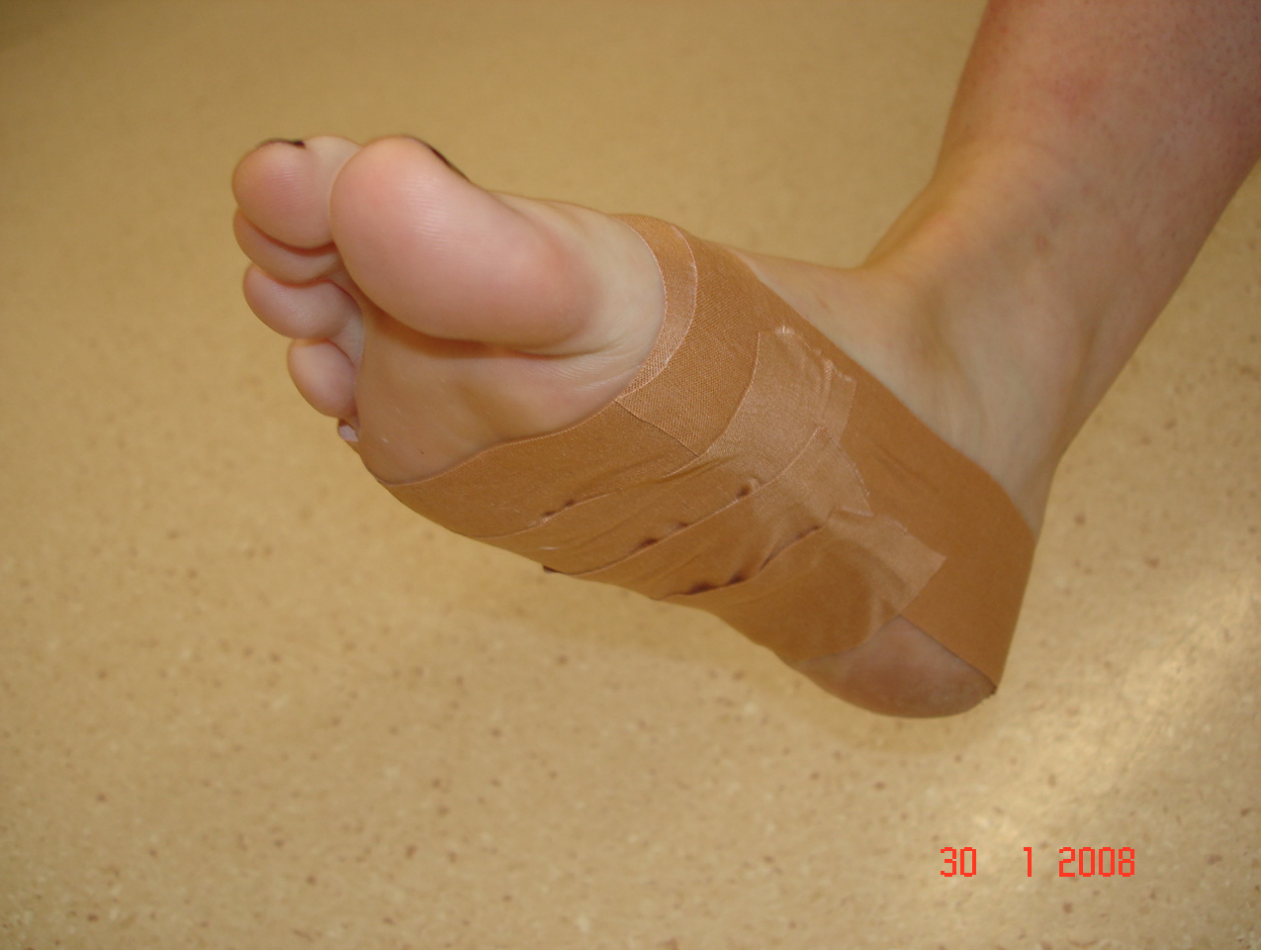
**Low-Dye taping technique**.

A standard LD taping technique was used (Figure 2), similar to that described by Vicenzino et al. [1]. Rigid Leukotape (38 mm) with a zinc oxide adhesive was applied while the subject was positioned in long-sitting with the foot in both talocrural and subtalar joint neutral, as palpated by the investigator. The foot was patted down with a dry towel prior to taping in order to maximise tape adherence. To ensure consistency, the same investigator carried out all taping procedures involved in the study, following a standardised protocol.

### Data Collection

**Figure 3:**
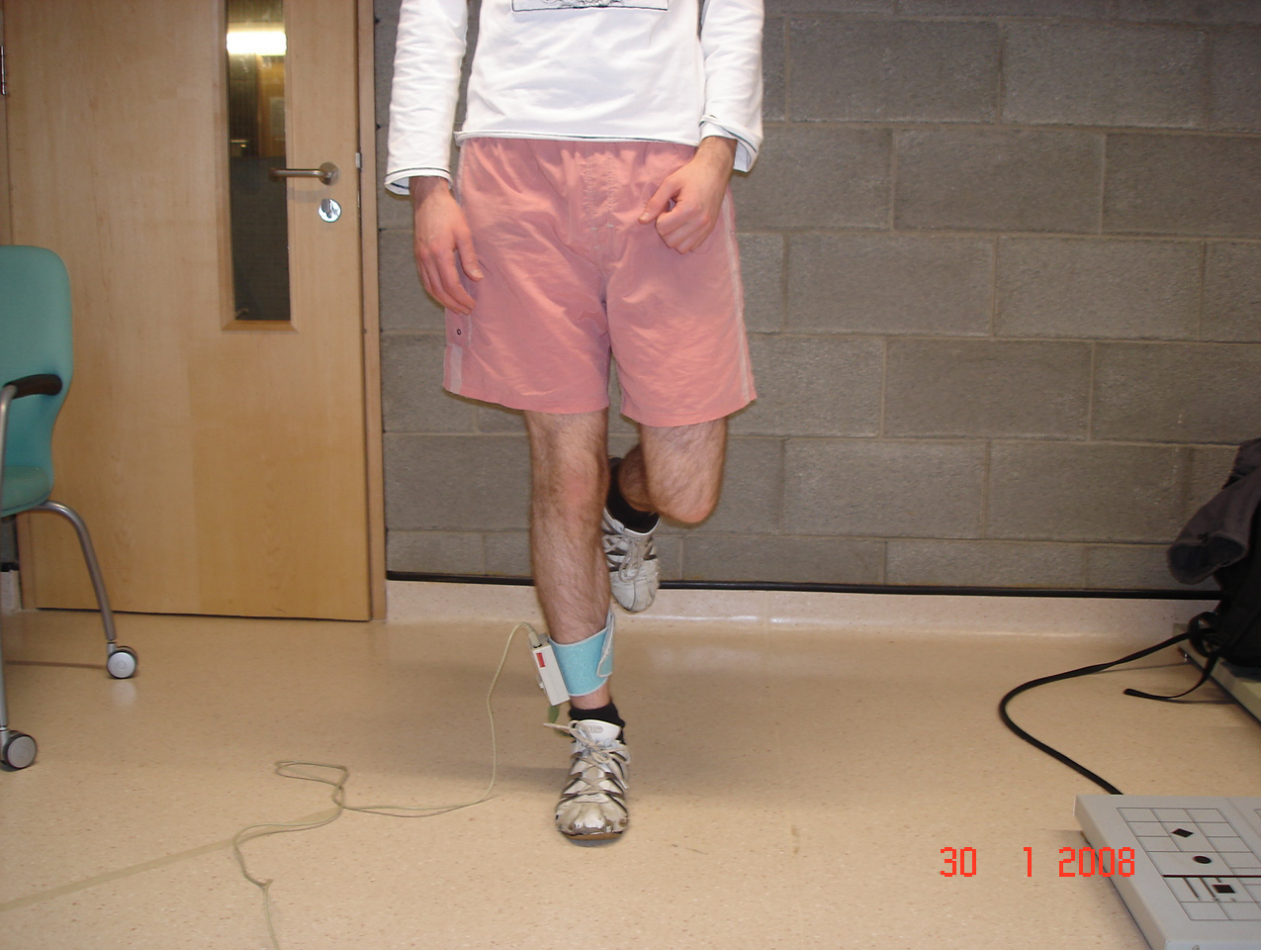
**Insole calibration**.

Prior to actual testing, step length of each subject was calculated during normal gait, as this measurement was required during subsequent pedometer use. A 10-minute rest was then provided, so that all subjects were relaxed on commencing the testing procedure. During this time, the investigator cut a new F-scan insole to suit the participant's shape of foot. The insole was then placed in the shoe of the foot to be taped, with the remainder of the 10-minute rest allowing time for the insole sensors to achieve a stable in-shoe temperature prior to testing [14]. Following this rest, insole calibration was performed as per manufacturers' guidelines. This involved the subject being asked to fully weight-bear through each foot for 1 second while in standing (Figure 3). The duration of 1 second was chosen as this best represented the duration of stance phase during gait in this subject population. Plantar pressure was recorded, at a frequency of 50 frames/second, over 10-metre walks along the flat floor surface of the testing laboratory. Standardised instructions were provided to all subjects i.e. to walk at a normal, comfortable pace, looking straight ahead. The same insole was used during each of the four walks performed throughout the session. Subjects initially performed one practice walk in order to familiarise themselves with the F-scan cuff unit and Velcro strap. The first 10-metre walk was then carried out, with un-taped plantar pressure data being recorded by the F-scan. Following this walk, the investigator applied the LD tape to the subject's foot. Subjects then repeated the 10-metre walk, with baseline-taped plantar pressure measurements being recorded. The exercise component of the study consisted of walking at a normal, self-selected pace, as has been employed in previous studies [9]. This took place along a flat, level, rectangular-shaped track outside the testing laboratory. 20 minutes was chosen as the exercise duration, as previous research suggests that LD tape has completely lost its effect on rearfoot motion (another measure of subtalar joint pronation) after 30 minutes of walking [9]. However, in order to investigate the possibility of the tape losing its effectiveness in less time, the exercise component was divided into two 10-minute walks, with plantar pressure being recorded following each walk (i.e. post exercise session 1, post exercise session 2). Overall duration of the walks varied slightly between participants, as each walk was terminated at the *end *of a lap of the track. The termination point closest to 10 minutes was chosen. The order of the testing session is shown in additional file 1.

As a loss in tape effectiveness could possibly be influenced by velocity and distance walked during exercise, the number of steps and distance walked in the two sessions were recorded by a pedometer. Calculation of the overall time and distance walked allowed velocity of the subjects to be assessed as a possible confounding factor. Subjects wearing shoe orthotics removed them from their shoes during the testing session, so as to ensure that the LD tape was the sole potential supplier of anti-pronatory force.

### Data Analysis

Each 10-metre walk recorded by the F-scan was visually represented as a movie by the Tekscan software. Mean data from 3 footfalls (i.e. stance phases) occurring mid-walk was used for analysis. Mid-walk data was chosen as acceleration and deceleration, associated with the beginning and the end of the walks respectively, could influence plantar pressure readings. Analysing the mean of 3 footfalls is adequately reliable (generalisability co-efficient 0.75) [12]. Prior to analysing the data, certain foot cells of each subject's movies were edited as per manufacturers' guidelines. This was employed where "shorts" had developed, i.e. where insole cells appeared to be loaded when they were not. For each movie, the foot was divided into 6 distinct grids, or "panes", representing the different anatomical regions of the foot. The location of these panes remained constant for analysis of a subject's four walks. However, different grids were used for each participant in order to accommodate variations in foot size.

For each of the 6 panes, mean peak plantar pressure over the middle 3 footfalls of a movie was calculated by the Tekscan software. The highest value recorded by a group of 4 adjacent cells over the entire footfall represented the peak pressure figure obtained [15].

### Statistical Analysis

Statistical analysis was performed using SPSS 15.0 for Windows. A one-way repeated measures analysis of variance (ANOVA) was employed to determine the significance of pressure variations in the 6 foot regions under the different conditions, i.e. un-taped, baseline-taped, post-exercise session 1 (PES1), and post-exercise session 2 (PES2). This parametric test was used as the data largely exhibited normal distribution, as evident from the Shapiro-Wilk test. Paired-sample t-tests were used to determine between which of the 4 testing conditions the possible differences occurred. Variations in time taken to complete the middle 3 footfalls in each of the 10-metre walks, as well as any variations in the components of the exercise sessions (duration, distance, velocity), were also assessed using paired-sample t-tests. Post-hoc analysis using Bonferroni multiple comparisons test was not performed as, following statistical advice, it was deemed too conservative a test for the small sample size of the study. Alpha levels were set at *p *< 0.05.

## Results

### Demographic Data

12 subjects (3 male, 9 female) met the inclusion criteria. Their mean (+/- SD) age was 25.92 (+/-12.32) years. Excessive pronation of the right foot was present in each of the subjects participating in the testing session, with bilateral excessive pronation occurring in 5 of the subjects. Thus, each subject's right foot was used for taping during the session. Mean navicular drop of the right foot was 12.13 mm.

### Adverse effects

There were no adverse effects of the tape or testing procedure on the subjects.

### Plantar Pressure Data

Mean peak plantar pressure data of the 12 subjects is shown in Table 1. Peak plantar pressure raw data is also detailed for each subject (see additional file 2).

**Table 1:** Mean (+/- SD) values for peak plantar pressure (measured in kilopascals).

	**Un-taped**	**Baseline-Taped**	**PES 1**	**PES 2**
**MF**	199.83 (+/- 47.96)	218.67 (+/- 71.24)	258.08 (+/- 98.56)	254.42 (+/- 90.98)
**LF**	193.17 (+/- 52.40)	167.83 (+/- 43.85)	193.58 (+/- 68.21)	206.67 (+/- 65.68)
**MM**	50.25 (+/- 13.43)	43.08 (+/- 15.96)	46.58 (+/- 16.26)	46.58 (+/- 24.99)
**LM**	72.58 (+/- 23.80)	83.08 (+/- 23.99)	81.67 (+/- 25.85)	84.25 (+/- 34.23)
**MR**	162.17 (+/- 32.65)	141.92 (+/- 27.34)	152.25 (+/- 19.52)	163.42 (+/- 23.80)
**LR**	144.33 (+/- 25.50)	131.92 (+/- 27.49)	139.25 (+/- 31.89)	151.42 (+/- 26.09)

Shown are the mean peak plantar pressure values for each of the four testing conditions for each region of the foot; medial forefoot (MF), lateral forefoot (LF), medial midfoot (MM), lateral midfoot (LM), medial rearfoot (MR) and lateral rearfoot (LR).

### Plantar Pressure Analysis

#### Forefoot

ANOVA (Table 2) showed that there was a variation in mean peak plantar pressure across the four testing conditions in the medial and lateral forefoot. Paired t-tests (Table 3) showed that there was a significant reduction in lateral forefoot peak plantar pressure (*p *= 0.039) after application of the tape (baseline-taped). However, pressure had significantly increased following each exercise session (*p *= 0.036 PES1, *p *= 0.004 PES2), and was once again similar to un-taped values. Each exercise session resulted in significantly higher medial forefoot peak pressure compared to un-taped (*p *= 0.015 PES1), (*p *= 0.014 PES2) and baseline-taped (*p *= 0.036 PES1), (*p *= 0.015 PES2).

**Table 2:** Results from one-way repeated measures ANOVA of each region of the foot.

**Foot Region**	***p*-value**
Medial Forefoot	0.002*
Lateral Forefoot	0.006*
Medial Midfoot	0.353
Lateral Midfoot	0.124
Medial Rearfoot	0.015*
Lateral Rearfoot	0.025*

* Result is statistically significant.

**Table 3:** Results from paired-sample t-tests.

**Pair Analysed**	**MF**	**LF**	**MM**	**LM**	**MR**	**LR**
UT – BT	0.283	0.039*	0.073	0.096	0.090	0.117
	
UT – PES1	0.015*	0.959	0.317	0.108	0.192	0.537
	
UT – PES2	0.014*	0.274	0.538	0.086	0.848	0.205
	
BT – PES1	0.036*	0.036*	0.142	0.637	0.128	0.165
	
BT – PES2	0.015*	0.004*	0.383	0.829	0.004*	0.004*
	
PES1 – PES2	0.742	0.188	1.000	0.643	0.010*	0.055

Shown are the statistical differences (*p*-value) in peak plantar pressure between each of the four testing conditions (un-taped, baseline-taped, post exercise session 1 and post exercise session 2) for each region of the foot; medial forefoot (MF), lateral forefoot (LF), medial midfoot (MM), lateral midfoot (LM), medial rearfoot (MR) and lateral rearfoot (LR).

*Result is statistically significant.

#### Midfoot

There were no differences in peak pressure between the four conditions. Although a trend towards a medial-to-lateral shift in pressure was present in the midfoot (Table 1), the changes were not statistically significant between any of the four testing conditions (Table 3).

#### Rearfoot

ANOVA (Table 2) again showed variations in mean peak plantar pressure across the four testing conditions were present in the medial and lateral rearfoot. Paired t-tests (Table 3) showed that peak plantar pressure values were significantly higher in the medial region following the second exercise session when compared to those at baseline-taped (*p *= 0.004) and post exercise session 1 (*p *= 0.010). Additionally, in the lateral rearfoot, peak plantar pressures after 20 minutes of exercise were also significantly higher (*p *= 0.004) than those at baseline-taped.

### Analysis of 10-metre walks

Paired-sample t-tests showed that the mean times taken to complete footfalls 3–5 in walks 3 and 4 (following each exercise session) were significantly less than in walk 1 (un-taped), indicating that subjects walked quicker during these latter walks (Table 4). Similarly, subjects also walked quicker during walk 4 than during walk 2 (baseline-taped). Raw data from the 10 metre-walks is detailed for each subject (see additional file 3).

**Table 4:** F-scan recorded walks

**Comparison**	***p*-value**
Walk 1/Walk 2	0.391
Walk 1/Walk 3	0.001*
Walk 1/Walk 4	0.003*
Walk 2/Walk 3	0.101
Walk 2/Walk 4	0.049*
Walk 3/Walk 4	0.323

Shown are the results from paired-sample t-tests comparing the times taken to complete footfalls 3–5 in each of the four walks recorded by the F-scan.

*Result is statistically significant.

### Measurements of exercise sessions

Paired-sample t-tests showed that the duration, distance walked, and velocity of walks did not differ between the first and the second exercise sessions (Table 5). Raw data from the exercise sessions is detailed for each subject (see additional file 4).

**Table 5:** Results from paired-sample t-tests for exercise sessions.

**Pair Analysed**	***p*-value**
Duration Session 1 -Duration Session 2	0.550
Distance Session 1 -Distance Session 2	0.153
Velocity Session 1 -Velocity Session 2	0.343

## Discussion

This exploratory study investigated the effect of LD taping immediately post-application, and again after two 10-minute exercise sessions, and found that the only immediate effect of the taping technique was a statistically significant reduction in peak plantar pressure in the lateral forefoot. Following 10 minutes of exercise, peak plantar pressure in the medial forefoot had significantly increased when compared to un-taped and baseline-taped. Furthermore, the initial reduction in lateral forefoot pressure was no longer present, i.e. peak pressure had returned to its un-taped value.

Following 20 minutes of taped exercise, medial forefoot peak plantar pressure still remained significantly higher than un-taped and baseline-taped values. Rearfoot pressure was also significantly higher compared to baseline-taped values. Midfoot recordings showed a trend towards a medial-to-lateral shift in pressure at baseline-taped, which was still present after 20 minutes of exercise.

### Initial effects

The initial decrease in lateral forefoot peak plantar pressure following taping is in accordance with the findings of Lange et al. [2], and Russo and Chipchase [7]. LD tape can induce restrictions in midtarsal joint motion, preventing moulding of the forefoot to the ground, and thus reducing plantar pressure in the region [2]. However, this mechanism of action is contradicted by the trend towards increased medial forefoot peak plantar pressure following taping found in this study.

Although no significant differences were present between un-taped and baseline-taped measurements in the midfoot and rearfoot, certain trends emerged which agree with results obtained by O' Sullivan et al. [16] and Lange et al. [2]. The reduction in rearfoot pressure with tape application may represent a controlling of overall foot hypermobility, more than an actual tape-induced restriction in subtalar joint pronation. The tape may be promoting rigidity of the foot, subsequently increasing load transfer from the rearfoot to lateral midfoot during the mid-stance phase of gait [2]. This is seen in the trend towards increased peak plantar pressure present in the lateral midfoot. Perhaps the non-significant increase in medial forefoot pressure could also be attributed to weight-transfer from the rearfoot, although neither Lange et al. [2], nor O' Sullivan et al. [16] reported increased medial forefoot peak plantar pressure with tape application, despite their reductions in rearfoot pressure.

Midfoot data demonstrated trends towards decreased and increased peak plantar pressure, for medial and lateral regions respectively, in comparison to un-taped measurements. This medial-to-lateral shift is representative of an anti-pronatory pressure change in the region, which is traditionally thought to be due to the LD tape providing a supinatory force at the medial plantar aspect of the foot [6]. These trends are in line with significant results obtained by Russo and Chipchase [7]. It is possible that the small sample size of the current study prevented these midfoot trends from achieving statistical significance.

### Effects post exercise

Following 10 minutes of exercise, mean peak plantar pressure in the lateral forefoot had returned to its un-taped level. Midfoot and rearfoot pressures were exhibiting similar regressions following their initial, tape-induced trends. This suggests a decline, or even loss, in tape effectiveness, possibly due to decreased tape tensile strength or reduced adhesiveness to the skin [3].

Peak plantar pressure in the medial forefoot had significantly increased after 10 minutes of exercise, following on from its trend towards increased pressure at baseline-taped. This indicates that for this region, the tape may actually be *increasing *its effect over time, something that no previous research has found. It is difficult to understand why medial forefoot peak plantar pressure increased with taped walking, as regressing data in the remaining regions would imply that the tape is losing any initial effect, and that load is no longer potentially being transferred to the region. As this is the first study to investigate LD tape's effect on plantar pressure over a timed period of exercise, no previously outlined hypothesis can be referred to.

Nonetheless, two potential mechanisms of action could be responsible. Considering that the tape appears to have a reduced influence on widespread plantar pressure after 10 minutes of activity, it is possible that the increase in medial forefoot pressure is due to *indirect *effects of the tape. Findings from research at the University of Queensland, Australia by Franettovich et al. in 2007 may hold particular relevance. Subjects in that study demonstrated decreased soleus activity during a 10-minute taped walk, which continued following removal of the augmented LD tape. These results suggest that the tape itself may induce carry-over effects in soft tissue structures. Likewise, Smith et al. [17] state that short-lived adaptations in soft tissue and neuromotor systems may persist following the removal of anti-pronatory tape. Perhaps the increased medial forefoot pressure after both exercise sessions could be due to a carry-over effect in activity of specific foot musculature, following initial facilitation from the tape. It is important to note that this hypothesised carry-over effect is occurring while the tape is still applied, and not after its removal, as mentioned previously. However, it does appear that the tape is exhibiting a reduced widespread effect as this stage. In any case, any neuromuscular adaptation that may be occurring at the medial forefoot is counter-productive in terms of reducing pronation. Excessive pronation contributes to the development of abnormalities of the first metatarsophalangeal joint, due to increased weight transmission medially across the foot [4]. Thus, the high medial forefoot pressure values present after 10 and 20 minutes of walking are not desirable, as they represent increased pronation.

A second hypothesis involves the influence of the exercise itself. Continued loading of the pronated foot during walking can lead to stress along medial joint capsules, ligamentous structures, and most importantly, tibialis posterior muscle [18]. This muscle, responsible for providing stability at the subtalar joint during activity, exhibits reduced efficiency in the pronated foot [18]. Regardless of the presence of LD tape, weight-bearing exercise sessions can lead to tibialis posterior lengthening and fatigue in pronated subjects [18], allowing the foot to fall into a more pronated, hypermobile position. Thus, perhaps the tape was ineffective during the exercise sessions, and any changes in plantar pressure distribution were solely exercise-induced. The absence of un-taped controls in the exercise sessions makes it difficult to determine whether continued increases in medial forefoot plantar pressure are due to indirect effects of the tape, or the exercise itself.

In addition to increased forefoot pressure, the other noteworthy finding following 20 minutes of exercise was that rearfoot peak plantar pressure had significantly increased compared to baseline-taped, with measurements now being similar to un-taped values. This, therefore, suggests a loss in the tape's initial anti-pronatory trend in the region, possibly as a result of mechanisms mentioned earlier.

In relation to the aims of the study, the main proposed mechanism behind LD taping is that it restricts rearfoot pronation, thereby reducing medial loading and increasing lateral loading through the foot [1,9]. The absence of any *significant *medial-to-lateral pressure shifts suggests that LD taping did not succeed in restricting pronation in the current study. Previous research has indicated short-lived effectiveness of LD taping on alternative measures of pronation; its effect on rearfoot motion lost after 30 minutes walking [9], navicular height improvements lost after a 10-minute jog [1]. In this asymptomatic population, LD tape's only initial effect, i.e. reduced lateral forefoot peak plantar pressure, was lost after a 10-minute walk. However, the tape may still possibly be maintaining an indirect effect on medial forefoot peak plantar pressure after 20 minutes of walking. Additionally, the trend towards a medial-to-lateral pressure shift present in the midfoot at baseline-taped is also still being maintained after the second exercise session, thus contrasting the pronatory pressure distribution evident in the medial forefoot.

### Limitations

The primary limitation is that the data obtained in the study exhibited high variability, as is commonly observed in studies examining plantar pressure using the F-scan [12,13]. To promote consistency, a strict protocol was implemented during F-scan use. The F-scan system is well correlated with force platform measures [12] and is adequately reliable [13], particularly when a mean of 3 steps is used to obtain the final pressure value [12]. In order to further limit variability, factors which could affect internal validity were taken into account. Velocity, duration, and distance walked were consistent between the two exercise sessions. However, in relation to the 10-metre walks, the mean duration of the middle 3 stances differed significantly between certain walks (Table 4). Therefore, in these cases, velocity cannot be eliminated as a confounding factor for peak plantar pressure values obtained [19]. To minimize error, one investigator carried out all taping involved in the study. However, the investigator's taping technique was not tested for intra-rater reliability. Immediate, post tape application pressure changes reported by Lange et al. [2] and O' Sullivan et al. [16] did not occur in the current study. Perhaps limited inter-rater reliability of LD taping techniques themselves may explain why. Russo and Chipchase [7] found that for midfoot and forefoot pressure measurements, inter-rater intraclass correlation coefficients between 0.28 and 0.69 were obtained for a standard LD taping technique.

Although each subject wore athletic trainers, footwear was not completely standardised. Hennig and Milani [20] reported that different shoe models significantly influence peak plantar pressures during running. However, in terms of clinical relevance, shoe type is likely to vary from one pronated individual to the next. Thus, enforcing identical footwear would have impeded external validity.

The sample size of the study was small, thus limiting the generalizability of the results. It is important to note that a larger sample size in the current study may have lead to the midfoot anti-pronatory trend gaining statistical significance, thus presenting the duration of the proposed anti-pronatory effectiveness of LD tape in a much more positive manner. Due to the exploratory nature of the study, a power calculation was not performed. Additionally, subjects were not randomly selected, but were a sample of convenience, and neither subjects nor investigators were blinded to the taping condition, as this was not feasible. Finally, in conjunction with similar studies, the present findings should be interpreted with caution given that no direct relationship between subtalar joint kinematics and plantar pressure has been established [2,7].

### Future Research

As this is the first study investigating the effectiveness of LD taping on plantar pressure distribution before and after exercise, additional similar studies are required. Larger sample sizes should be incorporated to enhance external validity. In light of the current findings, trials including un-taped controls in their exercise components, as well as further research examining muscle activity in the foot during taped exercise, are warranted. Furthermore, any potential proprioceptive capacity of LD tape should be investigated. Tape applied at the ankle has been shown to enhance proprioception [21], yet LD tape remains unexplored in this context. Perhaps proprioceptive input from the tape may have influenced subtalar joint position sense during the exercise sessions, and thus altered plantar pressure distribution.

### Clinical Relevance

In terms of clinical application, the primary use of LD tape is as a predictor for successful subsequent orthotic management [8]. The absence of significant widespread anti-pronatory plantar pressure alterations in the current study would arguably bring its aforementioned usage into question. From an intervention point of view, the current results suggest that LD tape does not reduce levels of pronation during a dynamic, weight-bearing activity such as walking. It must be acknowledged, however, that asymptomatic individuals were examined in the current study. Thus, the results cannot be directly extrapolated to the symptomatic population. It is also important to note that although the tape did not enforce anti-pronatory changes during exercise, this does not necessarily imply that the tape cannot limit movement into the *extreme *end range of pronation, where it appears the greatest amount of tissue stress occurs [3]. Therefore, the taping technique may still be beneficial in reducing the risk of subtalar joint-related injury [3].

## Conclusion

This exploratory study into the immediate and post-exercise effects of LD taping has some noteworthy findings. The only immediate post-application effect of LD tape was a reduction in peak plantar pressure in the lateral forefoot. This effect was lost after a 10-minute walk. Increased peak plantar pressure was noted in the medial forefoot after 10 and 20 minutes of exercise, indicating increased pronation. This increase may have been due to indirect effects of the tape, or perhaps as a result of the exercise itself. The midfoot demonstrated anti-pronatory trends in peak plantar pressure, i.e. a medial-to-lateral shift, following application of the tape. This trend was still present after 20 minutes of exercise, thereby suggesting a long-lasting anti-pronatory force in the region. Overall, significant widespread anti-pronatory changes in peak plantar pressure did not occur either on application of LD tape, or following two 10-minute walks. Thus, the results of this study, despite its small sample size, question the use of LD tape as an intervention for reducing excessive pronation at the subtalar joint. As this is the first to investigate LD tape's effect on plantar pressure before and after exercise, further similar trials with larger populations are required.

## Competing interests

The authors declare that they have no competing interests.

## Authors' contributions

DN was involved in conception and design of the study, data analysis and interpretation, as well as drafting and editing the final document for publication. NK was involved in conception and design of the study, data analysis and interpretation, as well as drafting and editing the final document for publication.

## Authors' information

DN graduated from the BSc Physiotherapy degree programme at the University of Limerick in 2008 with first-class honours. He is a member of the Irish Society of Chartered Physiotherapists (ISCP), and is currently practicing within the Irish Health Service Executive.

NK is a Lecturer in the Department of Physiotherapy at the University of Limerick, and a member of the ISCP. She holds a BSc (Physiotherapy), Postgraduate Diploma in Statistics and a PhD from Trinity College Dublin, and a Postgraduate Certificate in Teaching and Learning in Higher Education (NUIG).

## Pre-publication history

The pre-publication history for this paper can be accessed here:



## Supplementary Material

Additional File 1 **Order of testing session**. The table shows the components of the testing session and the order in which they were carried out. Click here for file

Additional File 2 **Raw peak plantar pressure data**. The data provided shows the raw peak plantar pressure data for each of the 12 subjects for the 10-metre walks at each of the 4 testing conditions: un-taped, baseline-taped (B. Taped), post-exercise session 1 (PES1), and post-exercise session 2 (PES2). Click here for file

Additional File 3 **Raw data from walks recorded by F-scan**. The data provided shows the time taken to complete stances 3–5 during the un-taped, baseline-taped, post-exercise session 1, and post-exercise session 2 walks recorded by the F-scan. Click here for file

Additional File 4 **Raw data from exercise sessions**. Duration, steps, distance walked, and velocity data of the exercise sessions. Click here for file
