# Supplementary material for: Effects of low-dye taping on plantar pressure pre and post exercise: an exploratory study
Source: BMC Musculoskelet Disord. 2009 Apr 21;10:40. doi: 10.1186/1471-2474-10-40 (PMC2676256; doi:10.1186/1471-2474-10-40)
Supplement: Additional File 1 — Order of testing session. The table shows the components of the testing session and the order in which they were carried out. [file 1471-2474-10-40-S1.doc]

**10-minute rest**

Step length calculated

F-scan insole cut

Strips of Low-Dye tape measured and cut

Walk 1

Un-taped plantar pressure recorded using F-scan

# Taping Procedure

Low-Dye tape applied by the investigator

Walk 2

Baseline-taped plantar pressure recorded using F-scan

Exercise session 1

10-minute walk outside laboratory

# Walk 3

Taped plantar pressure recorded using F-scan

Exercise session 2

10-minute walk outside laboratory

# Walk 4

Taped plantar pressure recorded using F-scan
