# Supplementary material for: Effects of low-dye taping on plantar pressure pre and post exercise: an exploratory study
Source: BMC Musculoskelet Disord. 2009 Apr 21;10:40. doi: 10.1186/1471-2474-10-40 (PMC2676256; doi:10.1186/1471-2474-10-40)
Supplement: Additional File 2 — Raw peak plantar pressure data. The data provided shows the raw peak plantar pressure data for each of the 12 subjects for the 10-metre walks at each of the 4 testing conditions: un-taped, baseline-taped (B. Taped), post-exercise session 1 (PES1), and post-exercise session 2 (PES2). [file 1471-2474-10-40-S2.doc]

| **Subject** | **Medial Forefoot (kPa)** | | | | **Lateral Forefoot (kPa)** | | | | **Medial Midfoot (kPa)** | | | |
| --- | --- | --- | --- | --- | --- | --- | --- | --- | --- | --- | --- | --- |
|  | Un-taped | B. Taped | PES1 | PES2 | Un-taped | B. Taped | PES1 | PES2 | Un-taped | B. Taped | PES1 | PES2 |
| 1 | 178 | 139 | 205 | 189 | 156 | 108 | 135 | 123 | 56 | 66 | 63 | 65 |
| 2 | 188 | 191 | 273 | 175 | 146 | 136 | 111 | 171 | 31 | 37 | 39 | 29 |
| 3 | 178 | 169 | 232 | 219 | 139 | 122 | 117 | 152 | 34 | 36 | 46 | 58 |
| 4 | 196 | 186 | 254 | 278 | 214 | 199 | 262 | 329 | 35 | 31 | 40 | 29 |
| 5 | 116 | 139 | 158 | 155 | 178 | 179 | 199 | 214 | 62 | 36 | 47 | 46 |
| 6 | 216 | 220 | 206 | 222 | 172 | 141 | 159 | 150 | 58 | 41 | 52 | 38 |
| 7 | 238 | 212 | 220 | 226 | 144 | 152 | 149 | 151 | 41 | 29 | 30 | 31 |
| 8 | 207 | 250 | 240 | 253 | 300 | 187 | 299 | 261 | 57 | 52 | 56 | 49 |
| 9 | 188 | 354 | 362 | 366 | 180 | 133 | 164 | 169 | 60 | 38 | 38 | 28 |
| 10 | 228 | 185 | 238 | 245 | 263 | 202 | 246 | 279 | 58 | 38 | 50 | 55 |
| 11 | 311 | 354 | 526 | 487 | 251 | 263 | 304 | 279 | 39 | 31 | 17 | 19 |
| 12 | 154 | 225 | 183 | 238 | 175 | 192 | 178 | 202 | 72 | 82 | 81 | 112 |
| **Mean** | 199.83 | 218.67 | 258.08 | 254.42 | 193.17 | 167.83 | 193.58 | 206.67 | 50.25 | 43.08 | 46.58 | 46.58 |

| **Subject** | **Lateral Midfoot (kPa)** | | | | **Medial Rearfoot (kPa)** | | | | **Lateral Rearfoot (kPa)** | | | |
| --- | --- | --- | --- | --- | --- | --- | --- | --- | --- | --- | --- | --- |
|  | Un-taped | B. Taped | PES1 | PES2 | Un-taped | B. Taped | PES1 | PES2 | Un-taped | B. Taped | PES1 | PES2 |
| 1 | 81 | 85 | 94 | 81 | 137 | 131 | 147 | 139 | 143 | 139 | 142 | 139 |
| 2 | 128 | 104 | 113 | 130 | 232 | 127 | 186 | 195 | 132 | 131 | 127 | 124 |
| 3 | 37 | 40 | 42 | 39 | 177 | 153 | 163 | 186 | 160 | 127 | 108 | 159 |
| 4 | 97 | 122 | 115 | 144 | 115 | 96 | 133 | 138 | 106 | 86 | 116 | 126 |
| 5 | 88 | 71 | 77 | 78 | 152 | 115 | 135 | 136 | 142 | 103 | 129 | 138 |
| 6 | 59 | 53 | 63 | 50 | 165 | 168 | 155 | 180 | 130 | 118 | 115 | 147 |
| 7 | 54 | 75 | 64 | 55 | 157 | 155 | 148 | 159 | 146 | 137 | 165 | 152 |
| 8 | 65 | 80 | 63 | 62 | 129 | 116 | 128 | 126 | 117 | 124 | 114 | 137 |
| 9 | 63 | 78 | 74 | 71 | 151 | 189 | 182 | 181 | 164 | 197 | 218 | 215 |
| 10 | 55 | 83 | 68 | 68 | 163 | 133 | 128 | 158 | 142 | 122 | 119 | 150 |
| 11 | 72 | 120 | 129 | 110 | 211 | 145 | 161 | 177 | 207 | 144 | 167 | 188 |
| 12 | 72 | 86 | 78 | 123 | 157 | 175 | 161 | 186 | 143 | 155 | 151 | 142 |
| **Mean** | 72.58 | 83.08 | 81.67 | 84.25 | 162.17 | 141.92 | 152.25 | 163.42 | 144.33 | 131.92 | 139.25 | 151.42 |
