# Supplementary material for: Effects of low-dye taping on plantar pressure pre and post exercise: an exploratory study
Source: BMC Musculoskelet Disord. 2009 Apr 21;10:40. doi: 10.1186/1471-2474-10-40 (PMC2676256; doi:10.1186/1471-2474-10-40)
Supplement: Additional File 3 — Raw data from walks recorded by F-scan. The data provided shows the time taken to complete stances 3–5 during the un-taped, baseline-taped, post-exercise session 1, and post-exercise session 2 walks recorded by the F-scan. [file 1471-2474-10-40-S3.doc]

| Subject | **Untaped (s)** | **Baseline**  **Taped (s)** | **Post Exercise Session 1 (s)** | **Post Exercise Session 2 (s)** |
| --- | --- | --- | --- | --- |
| 1 | 3.00 | 2.92 | 2.96 | 2.86 |
| 2 | 2.96 | 2.94 | 3.00 | 2.94 |
| 3 | 2.72 | 2.72 | 2.60 | 2.70 |
| 4 | 2.94 | 2.96 | 2.92 | 2.82 |
| 5 | 2.96 | 2.98 | 2.86 | 2.82 |
| 6 | 2.92 | 2.92 | 2.80 | 2.84 |
| 7 | 2.74 | 2.78 | 2.72 | 2.70 |
| 8 | 2.72 | 2.74 | 2.66 | 2.74 |
| 9 | 2.48 | 2.28 | 2.38 | 2.40 |
| 10 | 3.18 | 3.18 | 3.08 | 2.88 |
| 11 | 3.20 | 3.26 | 3.08 | 3.08 |
| 12 | 2.40 | 2.32 | 2.38 | 2.36 |
| **Mean** | 2.85 | 2.83 | 2.79 | 2.76 |
