# Supplementary material for: Effects of low-dye taping on plantar pressure pre and post exercise: an exploratory study
Source: BMC Musculoskelet Disord. 2009 Apr 21;10:40. doi: 10.1186/1471-2474-10-40 (PMC2676256; doi:10.1186/1471-2474-10-40)
Supplement: Additional File 4 — Raw data from exercise sessions. Duration, steps, distance walked, and velocity data of the exercise sessions. [file 1471-2474-10-40-S4.doc]

|  | Exercise session 1 | | | | Exercise session 2 | | | |
| --- | --- | --- | --- | --- | --- | --- | --- | --- |
| Subject | **Duration (s)** | **Steps** | **Distance walked (m)** | **Velocity (m/s)** | **Duration (s)** | **Steps** | **Distance walked (m)** | **Velocity (m/s)** |
| 1 | 590 | 1102 | 701 | 1.19 | 651 | 1215 | 773 | 1.18 |
| 2 | 607 | 1091 | 774 | 1.27 | 601 | 1083 | 768 | 1.26 |
| 3 | 581 | 1120 | 796 | 1.36 | 639 | 1253 | 891 | 1.38 |
| 4 | 597 | 1031 | 673 | 1.22 | 655 | 1194 | 780 | 1.17 |
| 5 | 611 | 1170 | 810 | 1.31 | 613 | 1159 | 802 | 1.29 |
| 6 | 643 | 1239 | 944 | 1.46 | 607 | 1201 | 915 | 1.50 |
| 7 | 643 | 1308 | 828 | 1.29 | 608 | 1268 | 803 | 1.31 |
| 8 | 634 | 1232 | 915 | 1.44 | 588 | 1172 | 871 | 1.46 |
| 9 | 638 | 1309 | 792 | 1.24 | 643 | 1315 | 800 | 1.24 |
| 10 | 604 | 1021 | 716 | 1.19 | 630 | 1115 | 782 | 1.25 |
| 11 | 597 | 1051 | 676 | 1.13 | 585 | 1163 | 748 | 1.29 |
| 12 | 606 | 1415 | 736 | 1.21 | 611 | 1378 | 716 | 1.17 |

|  | Exercise Session 1 | | | Exercise Session 2 | | |
| --- | --- | --- | --- | --- | --- | --- |
|  | **Duration** (s) | **Distance** (m) | **Velocity** (m/s) | **Duration** (s) | **Distance** (m) | **Velocity** (m/s) |
| Mean | 612.58 | 780.10 | 1.28 | 619.25 | 804.08 | 1.29 |
| **SD** | 21.53 | 87.02 | 0.11 | 23.78 | 59.24 | 0.11 |
